# Oxidative Stress and Neurotoxicity Biomarkers in Fish Toxicology

**DOI:** 10.3390/antiox14080939

**Published:** 2025-07-30

**Authors:** Grzegorz Formicki, Zofia Goc, Bartosz Bojarski, Małgorzata Witeska

**Affiliations:** 1 Institute of Biology and Earth Sciences, University of the National Education Commission, Podchorążych 2, 31-054 Krakow, Poland; grzegorz.formicki@uken.krakow.pl (G.F.); zofia.goc@uken.krakow.pl (Z.G.); 2 Department of Animal Biology and Environment, Faculty of Animal Breeding and Biology, Bydgoszcz University of Science and Technology, Mazowiecka 28, 85-084 Bydgoszcz, Poland; bbojarski@o2.pl; 3 Department of Animal Environment Biology, Institute of Animal Science, Warsaw University of Life Sciences, Ciszewskiego 8, 02-786 Warsaw, Poland

**Keywords:** antioxidant, redox, ROS, acetylcholinesterase, AChE, aquatic pollution

## Abstract

Exposure to xenobiotics causes pathophysiological changes in fish, including oxidative stress and neurotoxicity. Here, we describe the biochemical mechanisms underlying oxidative stress (i.e., redox imbalance) and the biochemical markers commonly used to assess its level. Neurotoxicity biomarkers used in fish include behavioral, histological, molecular, neurotransmitter-related, and enzymatic parameters, among which acetylcholinesterase (AChE) activity is the most commonly measured. We therefore also review the changes in AChE activity in fish exposed to common xenobiotics. In most cases, AChE activity decreased in a concentration- and time-dependent manner, although some studies reported no change or even an increase. We emphasize the relevance of all the parameters discussed in the context of fish toxicology studies.

## 1. Introduction

Oxidative stress and neurotoxicity often co-occur in fish exposed to toxic agents [1,2,3,4,5]. Therefore, this review aims to summarize and discuss the most relevant and recent literature on the use of oxidative stress parameters and neurotoxicity biomarkers in fish toxicological studies.

Model organisms used in monitoring and laboratory studies possess biological characteristics that are representative of a broader group of aquatic organisms, particularly those belonging to the same taxonomic units. Therefore, it can be assumed that aquatic pollutants inducing oxidative stress or altering AChE activity act in a similar manner across a wider range of aquatic species. Such changes affect animal behavior, motor activity, and metabolism, ultimately leading to a decline in the animals’ physiological condition. Based on this, it can be inferred that certain contaminants, through mechanisms involving oxidative stress and disruption of neural transmission, may influence relationships within ecosystems. For instance, they may increase mortality, reduce reproductive capacity, and affect various types of interactions within the environment. However, it is important to keep in mind that the outcomes of scientific research are significantly influenced by the conditions under which the studies were conducted. Our experience shows that different researchers apply varying fish stocking densities, which can influence water quality parameters, including the concentration of unionized ammonia (NH_3_)—a compound toxic to fish and whose concentration is often not reported in experimental studies. Therefore, general conclusions should be drawn with caution, particularly when they address broader phenomena than those directly investigated.

## 2. Indicators of Redox Imbalance in Fish Toxicology

In aerobic organisms, oxygen undergoes four electron reductions to produce adenosine phosphates, which leads to the formation of reactive oxygen species (ROS). Some ROS are essential to physiological regulation and processes. Free radicals are ROS with unpaired electrons. Primary free radicals are superoxide radical (O_2_^•−^), hydroxyl radical (OH^•^), hydroperoxyl radical (HOO^•^), alkyl peroxyl radical (LOO^•^), alkoxyl radical (LO^•^), and nitric oxide (NO^•^). They are unstable because they tend to form pairs with electrons of other compounds [6]. When oxidation and reduction reactions are unbalanced (i.e., redox imbalance), excessive amounts of free radicals are produced, which may lead to significant cellular damage [7,8].

The formation of free radicals may be assessed directly by the measurements of the O_2_^•−^ or H_2_O_2_ formation [9,10]. This method is rarely used in ecotoxicological studies because of the extremely short lifetime of the O_2_^•−^ anion or H_2_O_2_. Nonetheless, Falfushynska and Stoliar [11] indicated increased production of O_2_^•−^ in the gills and liver of crucian carp (*Carassius carassius*) obtained from contaminated waters. Free radicals cause lipid peroxidation, oxidation of DNA bases followed by DNA fragmentation, inhibition of enzymes, and damage to numerous structural compounds [12,13,14]. The products of oxidative damage of chemical molecules are direct evidence of the occurrence of reactive oxygen species in the organism. They are often used as markers of oxidative stress (Table 1). Indication of lipid peroxides as a level of thiobarbituric acid reactive substances (TBARS) belongs to the most popular methods.

Several studies reported increased levels of TBARS in different organs of fish from polluted water. A correlation also occurred between TBARS and the production of O_2_^•−^ in the liver and gills of common carp (*Cyprinus carpio*) [11]. On the other hand, in fish organs with high antioxidative capacity, increased activity of antioxidative enzymes may not be followed by increased TBARS levels [39]. Products of guanosine hydroxylation such as 8-hydroxy-2′-deoxyguanosine (8-OHdG), 8-oxo-7,8-dihydro-2′-deoxyguanosine (8-oxodG), and 8-hydroxyguanine (8-OHGua) indicate genotoxic effects of ROS. Analytical assays focused on the measurements of oxidative damage of guanosine indicated increased urinary levels of 8-OHGua in fish from polluted environments. However, most studies on the genotoxic effects of ROS in fish are conducted in controlled laboratory conditions. Other products of oxidative damage are protein carbonyls. Protein carbonyls may be a helpful indicator of oxidative stress under controlled laboratory conditions in the studies of model oxidative stress. Measurements of the level of protein carbonyls are rarely used in ecotoxicological studies. Despite their infrequent use in ecotoxicology, protein carbonyl measurements have revealed elevated levels in various tissues of gibel carp (*Carassius gibelio*) inhabiting an aquatic ecosystem influenced by a hydropower plant [40].

Organisms are protected against oxidative stress by nonenzymatic and enzymatic antioxidants. Nonenzymatic antioxidants provide electrons to free radicals, preventing oxidative damage. They include both endogenous and exogenous substances: vitamins (e.g., vitamin E, ascorbic acid), carotenoids, thiols (e.g., glutathione), uric acid, metallothioneins (MTs), polyphenols, flavonoids, and other compounds. Nonenzymatic antioxidants are non-specific and constitute the first defense against ROS [41]. Enzymatic antioxidants comprise specific antioxidative enzymes. Superoxide dismutase (SOD) converts O_2_^•−^ to H_2_O_2_. Manganese superoxide dismutase (MnSOD) expresses activity in mitochondria, while Cu,ZnSOD (copper-zinc superoxide dismutase) shows activity in cytosol and extracellular fluid [42]. Catalase (CAT) and glutathione peroxidase (GPx) catalyze the reduction of H_2_O_2_ to molecular oxygen and H_2_O. Their activity is usually coupled with SOD to prevent excessive H_2_O_2_ synthesis. Glutathione S-transferase (GST) also plays a vital role in preventing oxidative stress, which catalyzes the conjugation of harmful electrophiles to reduced glutathione (GSH) [42].

Both enzymatic and nonenzymatic antioxidants are often used as markers of oxidative stress. Nonenzymatic molecules are oxidized in the ROS reactions, so their levels may decrease during ROS excess. Metallothioneins (MTs) are induced mainly by zinc, cadmium, copper, and lead and are also engaged in metabolizing many other metals. Another essential function of MTs is protection against free radicals, where their methionine serves as a free radical scavenger [43]. Ecotoxicological studies mainly focus on MTs in the context of metal exposure and/or metal induction of oxidative stress. It was reported that fish from metal-contaminated environments showed increased MT gene expression, accompanied by elevated expression of antioxidative enzymes [44]. Increased MT concentration, oxidative damage, and enhanced activity of enzymatic antioxidants in the liver and gills of pelagic fish from metal-contaminated sites have also been documented [45].

Glutathione is one of the most studied molecules in fish subjected to oxidative stress. Most studies report increased concentrations of GSH in fish liver, kidneys, and gills from contaminated environments [9,46,47]. Increased GSH may suggest activating the antioxidative system or the phase II biotransformation reactions. The oxidized to reduced glutathione GSSG/GSH ratio may be used as the tissue redox state indicator. The relationship between GSSG/GSH and environmental contamination was found in crucian carp (*Carassius carassius*): the fish from contaminated water had higher GSSG/GSH than those from relatively clean water [48].

Adaptation of the cells to increased ROS production includes increased expression of genes encoding antioxidative enzymes and an increase in enzyme activity. Specific enzyme activity against ROS provides a valuable indicator of the redox balance in environmental studies. It has been shown that the activity of antioxidative enzymes (SOD, CAT, GR—glutathione reductase, GPx) is significantly correlated with oxidative damage products (TBARS, protein carbonyls) in the liver of sea trout (*Salmo trutta*) from the Słupia River (Poland) at different developmental stages [49]. Another study demonstrated differences in GPx gene expression in common carp (*Cyprinus carpio*) inhabiting two distinct water reservoirs in India [44].

### Organic Pollutants and Redox Imbalance

The influence of organic pollution on redox balance is complex. It mainly depends on the chemical properties of the compounds and their metabolism in fish organisms. Most of the organic pollutants affect the redox balance by several different mechanisms. A good example is the redox imbalance caused by ammonia. It occurs in two forms, NH_4_^+^ and NH_3_, which can be converted into each other. Exposure to ammonia causes the formation of ROS such as O_2_^•−^, OH^•^, H_2_O_2_, and ROO^•^ [50]. In estuarine teleost European sea bass (*Dicentrarchus labrax*), exposure to ammonia caused ROS formation and MDA accumulation in the brain, liver, gills, muscles, and kidneys. This effect was accompanied by the increased activity of SOD, CAT, GPx, and GR and increased concentration of GSH. However, these effects showed different dynamics in different tissues [50], which suggests different modulation of response to ammonia and free radicals in various tissues. Several mechanisms participate in ammonia-induced oxidative stress. First, and probably the most important to fish, is gill damage and subsequent hypoxia. Hypoxia shifts the mitochondrial redox balance toward a more reduced state, which may increase ROS production [7,51]. During hypoxia, the biosynthesis of nuclear factor-κb (NF-κb), an essential transcriptional regulator of proinflammatory factors, increases in fish gills [52]. Upregulation of proinflammatory cytokines TNF-α and IL-1β was also reported in other fish organs after exposure to ammonia [53,54]. Generally, inflammation is induced by free radicals, although some ROS are released by immune cells during the inflammation [55]. It may be expected that the immune system generates significant amounts of ROS in hypoxia. Ammonia has also influenced the expression of genes controlling redox balance. In great blue-spotted mudskippers (*Boleophthalmus pectinirostris*), exposure to ammonia resulted in the downregulation of the nuclear factor erythroid 2-related factor 2 (NRF2) [54]. It is known that the downregulation of NRF2 causes the downregulation of downstream genes encoding SOD, CAT, and GPx and the subsequent redox imbalance. Notably, glyphosate-based herbicides induce the exact mechanism studied in the liver tissue of common carp [17]. Another mechanism of oxidative stress induction by ammonia occurs in fish brains. Ammonia overstimulates the NMDA receptor. This causes a large influx of Ca^2+^ into the neural cells, followed by the generation of ROS and excitotoxicity [56].

Organic compounds undergo biotransformation by cytochrome P450 enzymes (CYPs). In fish, the CYP1 subfamily is expressed, and CYP1A is often used to indicate exposure to organic pollution [57]. Induction of CYP1A activates mixed-function oxygenases (MFOs), which catalyze the oxygenation of xenobiotics by single oxygen addition [58]. Redox reactions leading to the biotransformation of xenobiotics cause the formation of ROS. One of the most studied monooxygenases in fish is ethoxyresorufin-O-deethylase (EROD). Fish organs, mainly gills and liver, exposed to organic xenobiotics show increased activity of EROD and an increased rate of free radicals’ formation [59]. The participation of CYPs in oxidative stress induction in fish was confirmed by the studies on the toxicity of different organic compounds, including pyrethroid pesticides, hydrocarbons, chlorinated hydrocarbons, dioxins, organophosphates, and carbamates [58,59,60,61,62,63].

Other mechanisms mediating the induction of oxidative stress by organic xenobiotics are disturbances in mitochondrial electron transport, inhibition of enzymes, and depletion of nonenzymatic antioxidants. An example of a pesticide that induces oxidative stress by inhibiting electron transport in mitochondria is fenpyroximate [61]. Exposure to fenpyroximate caused increased activity of SOD, GPx, and CAT in the gill cell line and liver tissue of flounder (*Paralichthys olivaceus*) [64]. Electron transport in mitochondria may be interrupted by enzyme inhibition. Chlorothalonil, an organochlorine pesticide, inhibits the activity of NADPH oxidase by binding to its SH groups. This was found to cause disturbances in electron transport in mitochondria and the generation of free radicals in macrophages of striped bass (*Morone saxatilis*) [61]. Other mechanisms of oxidative imbalance may be related to the depletion of antioxidant levels. Pesticides may cause GSH depletion through two mechanisms, i.e., generating free radicals and conjugating with GSH in the second detoxification phase. Both effects may lead to oxidative stress. Such a mechanism of induction of oxidative stress was indicated after exposure of rainbow trout to organophosphates, methylparathion, and diazinon. Oxidative imbalance also occurred in the rainbow trout’s liver, gills, and muscles [65]. An interesting mechanism occurs in fish exposed to paraquat, a bipyridyl herbicide. Paraquat is reduced to its cation radical by glutathione reductase (GR); next, the radical is neutralized by the conjugation to GSH. This way, the radical chain reaction is stopped, although it leads to the depletion of GSH and lower protection against ROS. Moreover, GSH depletion leads to increased GR activity and a generation rate of paraquat cation radicals. The occurrence of oxidative stress accompanied by depletion of GSH and increased activity of GR seem to confirm the above mechanism in Nile tilapia (*Oreochromis niloticus*) [61].

The effects of toxic agents on fish oxidative stress parameters are presented in Table 2. In the cited studies, the most frequently determined parameters of oxidative stress in fish were glutathione; enzymatic antioxidants: SOD, CAT, GPx, and GST; and oxidative damage products: MDA and TBARS. Only in approximately 15% of the analyzed cases were no changes in the mentioned oxidative stress parameters noted. In comparison, in 85% of cases, their increase and/or decrease in activity/concentration under the influence of various toxicants was reported. In particular groups of substances, a diverse biological response was observed in fish, probably due to the mechanism of action of the antioxidant barrier, which may be species-specific. The results (Table 2) show that most aquatic pollutants induced oxidative stress in fish, which is indicated by increased levels of oxidation products (e.g., TBARS, MDA, and LPO). Various effects on antioxidant defense were observed: an increase, sometimes accompanied by no symptoms of oxidative stress, indicating efficiency; or a decrease, suggesting a breakdown of the antioxidant system. These effects were related to the toxicant’s concentrations.

Differences in individual research results may also be related to the differences in experimental conditions (water parameters, exposure duration, etc.). Despite discrepancies in the research results and difficulties in determining changes typical for given substances and/or fish species, the enzymatic and nonenzymatic parameters discussed in this review are commonly used in fish toxicology (both in field and laboratory studies) and provide valuable information about the organisms’ status.

## 3. Neurotoxicity Biomarkers in Fish Toxicology

The development and use of neurotoxicity biomarkers is an essential issue in toxicology because of numerous emerging pollutants, such as microplastics, pharmaceuticals, and other novel chemical compounds, as well as “old” contaminants. Metals and pesticides exhibit neurotoxic potential, leading to diverse deficits in neural systems representing varying levels of evolutionary advancement [109]. Neurotoxicity in fish may result in disturbed schooling, migration, spatial distribution, feeding, reproduction, and predator avoidance. Neurotoxicity biomarkers used in fish include histopathological [110,111] and morphometric [112] evaluation of the brain, various behavioral endpoints [113,114,115], expression of marker genes associated with neuron development and growth [116], and the levels of neurotransmitters in brain [117,118]. However, changes in the activity of cholinesterases (ChEs) are the most often used biomarkers of aquatic pollution because these enzymes are frequent targets for toxic effects of contaminants such as insecticides or metals [119]. This group of enzymes includes, among others, acetylcholinesterase (AChE), butyrylcholinesterase (BChE), propionylcholinesterase (PChE), and carboxylesterase (CbE). These enzymes are found in various tissues and are involved in detoxification processes in fish. The sensitivity of BChE, PChE, and CbE to different pesticides and other compounds can vary not only between fish species but also between different tissues within the same species. Moreover, their precise physiological roles remain unclear. Among cholinesterases, AChE is the most commonly used biochemical biomarker of neurotoxicity. AChE activity is usually measured in the brain and muscles, and also in the whole body (in the case of embryos and larvae), or in other organs such as liver and gills [120]. Brain or muscle AChE inhibition significantly correlated with reductions in spontaneous swimming and feeding activity in fish, and threshold concentrations for sublethal neurochemical and behavioral endpoints were similar [121,122].

### 3.1. Acetylcholinesterase as a Neurotoxicity Biomarker

Acetylcholinesterase (AChE), a serine carboxylesterase, is an important component of cholinergic synapses that terminates impulse transmission by rapid hydrolysis of the neurotransmitter acetylcholine (ACh). AChE is a very efficient catalyst that accelerates the substrate hydrolysis by more than eight orders of magnitude [123]. An AChE catalytic action includes a two-stage reaction: acylation and deacylation [124]. Although the primary function of AChE is to terminate neural transmission, it was also found that AChE plays a role in neural development [125]. AChE occurs in all tissues and is most abundant in the brain and muscles. AChE inhibition increases ACh concentration and neurotransmitter action [126], causing cognitive and behavioral disturbances. AChE activity is a sensitive biomarker of fish exposure to toxic agents [127]. However, its use requires an understanding of natural fluctuations. Seasonal variation in AChE activity has been observed in *Cnesterodon decemmaculatus*, with the highest values recorded in summer and a marked decrease in winter, likely associated with water temperature differences [128]. Seasonal changes in AChE activity and sensitivity to pesticides (glyphosate and chlorpyrifos) were also reported by other researchers for the same fish species [129].

Table 3 summarizes the effects of various toxic agents on AChE activity (100 papers containing 119 data points; in some studies, the toxicity of more than one compound was evaluated). Most data (45%) concern pesticides (35%—insecticides, 8%—herbicides, 2%—fungicides). Other groups of data concern pharmaceuticals and disinfectants (12%), elements (10%), microplastics (7%), and nanoparticles (6%). The remaining 18% of data describe the effects of other compounds such as antifouling agents, cosmetic components, aromatic hydrocarbons, cyanotoxins, plastic components, and others. Most experiments (44%) were conducted on embryos, larvae, or adults of *Danio rerio*.

Analysis of the methods of AChE activity measurements (in 100 papers) revealed that most authors (66%) used the method developed and described by Ellman et al. [130], 23% used commercial biochemical kits, 4% used other methods, and 7% did not specify the method used. Spectrophotometric cuvettes (16%) were often replaced by microplates (27%), and 57% of authors did not specify the reading method (but commercial kit users presumably used microplates; thus, microplates were probably used in most studies). However, it has been shown that copper, zinc, cadmium, and mercury affect the results of AChE measurements in vitro, likely due to their reactions with the products of Ellman’s reaction [131]. This poses a challenge to obtaining accurate data, particularly when assessing the in vitro neurotoxicity of these metals.

**Table 3:** The effects of toxic agents on brain (or whole body—in embryos and larvae) acetylcholinesterase (AChE) activity in fish. The original values are given in parentheses and expressed in the following units: ° mg/L * µg/L ^#^ ng/L ^××^ mg/kg feed ^×××^ g/kg feed ^$^ µg/kg sediment “” mg/L algal extract ^@^ particles/L.

GCS	Fish Species	Toxic Agent	Concentration [µmol]	Exp. dur. [d]	AChE Activity	Reference
M	*D. rerio*	microplastic	(10,000 ^@^)	5	↓	Xue et al. [132]
M	*D. rerio* embryos	microplastic	(0.1–10 °)	4	↓	Suman et al. [133]
M	*D. rerio* embryos	microplastic	(0.1–3 °)	5	↓	Martin-Folgar et al. [134]
M	*O. javanicus*	microplastic	(0.5–5 °)	21	↑	Usman et al. [135]
M	*O. mossambicus*	polypropylene microplastic	(100 ^××^)	14	–	Jeyavani et al. [136]
			(500 ^××^)		↓	
			(1000 ^××^)		↓	
M	*O. niloticus*	microplastic	(100 °)	21	↓	Yang et al. [137]
M	*D. rerio*	microplastic	(2 °)	30	↑	Santos et al. [138]
E		copper (as CuSO_4_•5H_2_O)	0.157 (25 *)		↑	
M	*D. labrax*	microplastic	(0.26 ° or 0.69 °)	4	↓	Barboza et al. [72]
E		mercury (as HgCl_2_)	0.037–0.059 (0.010 ° or 0.016 °)		↓	
E	*A. testudineus*	chromium VI (as CrO_3_)	27.503 or 55.006 (2.75 or 5.5 °)	72	↓	Kumar et al. [139]
E	*C. decemmaculatus*	arsenic (as NaAsO_2_)	3.849–38.488	4	–	González Núñez et al. [140]
			(0.5–5 °)			
E	*D. rerio*	chromium III (as CrCl_3_•6H_2_O)	6.315 (1 °)	5	↓	Xu et al. [141]
E		chromium VI (as (K_2_Cr_2_O_7_)	3.400 (1 °)		↓	
E	*D. rerio*	aluminum	203.840 (5.5 °)	15	↓	Alves et al. [142]
E	*D. rerio* embryos	mercury (as HgCl_2_)	36.830 (10 °)	1	–	Henriques et al. [143]
				2–4	–	
			368.297 (100 °)	1	–	
				2–4	↓	
E	*D. rerio* embryos + larvae	antimony (as K_2_Sb_2_C_8_H_4_O_12_	299.460–1197.838	2	↓	Xia et al. [144]
		• 3 H_2_O)	(200–800 °)			
E	*H. molitrix* larvae	mercury (as HgCl_2_)	0.004–0.037	14	↓	Wang et al. [145]
			(1–10 *)			
E	*O. niloticus*	aluminum (as Al_2_(SO_4_)_3_	0.003–0.009	14	↑	Oliveira et al. [146]
			(1 or 3 *)			
E	*O. niloticus*	titanium (as TiO_2_)	0.626 or 1.252 (0.05 or 0.1 °)	7–14	–	Abegoda-Liyanage and Pathiratne [147]
N		titanium (as TiO_2_ NPs)			–	
N	*D. rerio*	selenium NPs	6.331 or 126.629 (0.5 or 10 °)	4	↓	Fan et al. [148]
N	*D. rerio*	silver NPs	9.271 (1 °)	4	–	Marinho et al. [149]
			0.028–0.046 (3–5 *)		↓	
N	*O. mykiss*	graphene nanoflakes	(4 °)	36	–	Jakubowska-Lehrmann et al. [150]
N		graphene oxide				
N		reduced graphene oxide				
N		silicon carbide nanofibers				
F	*D. rerio*	paclobutrazol	34.038 (10 °)	4–14	↓	Guo et al. [151]
F	*D. rerio*	thifuzamide	0.360 (0.19 °)	6	↓	Yang et al. [152]
			3.598 or 5.397 (1.9 or 2.85 °)			
F	*D. rerio* embryos	mancozeb	0.0009 (0.5 *)	4	↓	Vieira et al. [153]
			0.009 (5 *)		–	
			0.092 (50 *)		↓	
H	*D. rerio* embryos	Roundup^®^	1.479 (0.25 °)	2	↓	Ames et al. [154]
		glyphosate				
H	*D. rerio* larvae	Roundup^®^	0.028 (4.8 *)	5	↑	Pompermaier et al. [155]
H	*D. rerio* larvae	haloxyfop-p-methyl	0.532–1.065	4	↑	Liu et al. [156]
			(0.2–0.4 °)			
H	*O. niloticus*	pendimethalin	1.848 (0.52 °)	28	↓	Hamed and El-Sayed [157]
H	*P. lineatus*	Roundup^®^	5.915 or 29.574	4	↓	Modesto and Martinez [2]
			(1 or 5 °)			
H	*C. carpio*	glyphosate	20.701 (3.5 °)	21	↓	Zhang et al. [158]
I		chlorpyrifos	0.071 (25 *)		↓	
H	*T. nilotica*	diuron	4.290 (1 °)	1	↓	El-Nahhal [159]
I		Nemacur^®^	0.330–6.593 (0.1–2 °)		↓	
I		malathion	0.303–6.054 (0.1–2 °)		↓	
H	*D. rerio*	DMA^®^ 806 BR (Fipronil)	0.134 (63.5 *)	4	↑	Viana et al. [160]
I		Regent^®^ 800 WG (2,4-D)	(447 *)		↑	
I	*C. auratus gibelio*	trichlorfon	1942.2–7768.8	0.5–4	↓	Lu et al. [161]
			(0.5–2 ^×××^)			
I	*C. carpio*	diafuran	14.267–48.802	4	↓	Golombieski et al. [162]
	*C. idella*		(1–3 °)			
	*A. nobilis*					
I	*C. carpio*	chlorpyrifos	0.066 or 0.131 (23 or 46 *)	14	↓	Pala et al. [163]
I	*C. carpio*	λ cyhalothrin	0.0003 or 0.0006 (0.14 or 0.28 *)	15–45	↓	Chatterjee et al. [164]
I	*C. macropomum*	malathion	22.097 (7.3 °)	4	–	de Souza et al. [90]
I	*C. macropomum*	trichlorfon	1.010 or 1.670 (0.26 or 0.43 °)	1–4	↓	Duncan et al. [165]
I	*C. punctatus*	triazophos	0.011 or 0.022 (3.4 or 6.8 *)	4	↓	Singh et al. [166]
I		deltamethrin	0.007 or 0.0014 (0.36 or 0.72 *)		↓	
I	*C. umbla*	chlorpyrifos	156.880 (55 °)	1	–	Kirici [111]
				4	↓	
			313.760 (110 °)	1	↓	
				4	↓	
I	*Clarias batrachus*	thiamethoxam	23.756 or 47.513 (6.93 or 13.86 °)	45	↓	Mukherjee et al. [167]
I	*D. rerio*	chlorpyrifos	0.003 (1.16 *)	5	↓	Zhang et al. [168]
I		cyfluthrin	0.016 or 0.033 (7.06 or 14.12 *)		↓	
I	*D. rerio*	dinotefuran	0.989 (0.2 °)	28	↓	Ran et al. [169]
			4.946 (1 °)		↓	
I	*D. rerio*	imidacloprid	0.0006 (0.15 *)	4	–	Guerra et al. [170]
			0.059 or 0.176 (15 or 45 *)		↓	
I	*D. rerio*	imidacloprid	0.0002–0.078 (0.05–20 *)	14–35	–	Zhang et al. [171]
I		thiamethoxam	0.0002–0.069 (0.05–20 *)		↓	
I	*D. rerio*	sulfoxaflor	3.138–12.659 (0.87–3.51 °)	4	↑	Benli and Celik [172]
I	*D. rerio*	methomyl	3.082–143.641 (0.5–23.3 °)	6	↓	Jablonski et al. [173]
I	*D. rerio* embryos	chlorphoxim	7.513–22.540 (2.5–7.5 °)	4	↓	Xiong et al. [174]
I	*D. rerio* larvae	fenpropathrin	0.046–0.183 (0.016–0.064 °)	4	↑	Yu et al. [175]
I	*D. rerio* larvae	isoprocarb	5.175–12.937 (1–2.5 °)	6	↓	Wang et al. [176]
I	*G. affinis*	chlorpyrifos	0.847 (0. 297 °)	4	↓	Kavitha and Rao [177]
I	*G. affinis*	cypermethrin	4.8 × 10^−7^ or 1.5 × 10^−5^	7	↓	Touaylia et al. [178]
			(0.2 or 6.25 ^#^)			
I	*G. affinis*	carbofuran	0.863 or 0.701 (0.191 or 0.255 °)	15–40	↓	Rouachdia et al. [179]
I	*H. fossilis*	chlorpyrifos	0.257 or 0.548 (0.09 or 0.192°)	7–30	↓	Mishra et al. [180]
I	*J. multidentata*	cypermethrin	9.610 × 10^−5^ or 9.610 × 10^−4^	4	–	Bonansea et al. [122]
			(0.04 or 0.4 *)			
I		chlorpyrifos	0.001 or 0.011 (0.4 or 4 *)		–	
I	*O. latipes*	diazinon	0.033 or 0.066 (10 or 20 *)	122	↓	Flynn et al. [181]
I	*O. mykiss*	chlorpyrifos	6.413 or 12.830	1–4	–	Topal et al. [182]
			(2.25 or 4.5 °)			
			0.021 (7.25 *)	1–2	–	
			0.021 (7.25 *)	3–4	↓	
I	*O. mykiss*	chlorpyrifos	0.006 (2 *)	7	–	Mehtabidah et al. [183]
			0.006 (2 *)	14–21	↓	
			0.011 (4 *)	7–21	↓	
			0.017 (6 *)	7–21	↓	
I	*O. mykiss*	phosmet	0.016 (5 *)	1–2	–	Muhammed and Dogan [184]
			0.016 (5 *)	3–4	↓	
			0.079 or 0.158 (25 or 50 *)	1–4	↓	
I	*O. mykiss* larvae	chlorpyrifos	0.0009 (0.3 *)	21	–	Weeks Santos et al. [185]
			0.009 (3 *)		↓	
I	*O. niloticus*	chlorpyrifos	14.251–42.753	30	↓	Oruç [3]
			(5–15 *)			
I	*O. niloticus*	malathion	4.313 (1.425 °)	1	↑	Amin et al. [186]
				2	↓	
I		chlorpyrifos	0.357 (0.125 °)	1	↑	
				2	↑	
I		λ-cyhalothrin	0.009 (0.0039 °)	1	–	
				2	↓	
I	*O. niloticus*	carbofuran	1.112 (0.246 °)	30	↓	Hamed et al. [187]
I	*P. lineatus*	fipronil	12.581 (5.5 °)	15	–	Santillan Deiú et al. [188]
			0.188 (82 ^$^)		↓	
I	*R. quelen*	trichlorfon	42.728 (11 °)	21	↓	Baldissera et al. [189]
Pd	*Corydoras paleatus*	triclosan	0.653 (189 *)	2	↓	Sager et al. [190]
Pd	*D. rerio*	fluoxetine	0.016–0.052 (5–16 ^#^)	4	↓	Orozco-Hernández et al. [191]
Pd	*D. rerio*	fluoxetine	0.003–0.0323 (0.1–10 *)	21	–	Correia et al. [192]
Pd	*D. rerio* embryos + larvae	cloramine T	70.286 (16 °)	4	–	Rivero-Wendt et al. [193]
			140.573 (32 °)		–	
			281.146 (64 °)		↓	
			562.291 (128 °)		↓	
Pd	*D. rerio* embryos + larvae	2, 5-dichloro-1, 4-benuinone	1.130 (0.2 °)	4	–	Chen et al. [194]
			2.260 or 3.390 (0.4 or 0.6 °)		↓	
Pd	*Danio rerio*	metformin	0.009–0.310 (1–40 *)	120	↓	Elizalde-Velázquez et al. [195]
Pd	*Danio rerio*	sertraline	3.266 (1 °)	28	–	Yang et al. [196]
			32.655 or 326.552 (10 or 100 °)		↑	
Pd	*Danio rerio*	nortriptyline	0.003–1.898 (0.88–500 *)	7	↓	Oliveira et al. [197]
Pd	*Danio rerio* embryos	moxidectin	0.002–0.008 (1.5–5 *)	4	–	Muniz et al. [198]
Pd	*Danio rerio* embryos + larvae	sertraline	3.266–326.552 (1–100 °)	10	–	Yang et al. [152]
Pd	*Gambusia affinis*	gestodene	1.42 × 10^−5^ (4.4 ^#^)	60	↓	Tan et al. [199]
			0.0012 (378.7 ^#^)		↑	
Pd	*Oreochromis mossambicus*	triclosan	0.452–3.613 (0.131–1.046 °)	4	↓	Deepika et al. [200]
Pd	*Oreochromis niloticus*	synthetic progesterone	0.636–2.544 (0.2–0.8 °)	4	↓	Rocha et al. [201]
Pd	*Rhamdia quelen*	ciprofloxacin	0.003 (1 *)	28	–	Carvalho et al. [202]
			0.030 or 0.302 (10 or 100 *)		↓	
O	*A. testudineus*	naphthalene	32.769–39.011 (4.2–5.0 °)	3	↓	Nayak and Patnaik [203]
O	*C. carpio*	ammonia NH_3_	1802.701 (30.7 °)	4	↓	Molayemraftar et al. [204]
O		nitrite NO^2-^	3341.304 (153.7 °)			
O	*C. gariepinus*	benzene	9.76 × 10^−6^ (0.762 ^#^)	30	↓	Sayed et al. [68]
O		toluene	0.0003 (26.614 ^#^)		–	
O		xylene	0.0011 (89.403 ^#^)		–	
O	*C. gariepinus*	burnt tyre ash	(0.56–2.24 °)	28	↓	Iheanacho et al. [205]
O	*C. mrigala*	phenol	24.652–73.956	7–28	↓	Muthukumaravel et al. [206]
			(2.32 or 6.96 °)			
O	*D. rerio*	tributylin	3.24 × 10^−5^ (10 ^#^)	42	–	Li and Li [207]
			0.0003–0.001 (100–300 ^#^)		↓	
O	*D. rerio*	bisphenol A	0.001–0.007 (0.22–1.5 *)	4	↑	Heredia-Garcia et al. [208]
O	*D. rerio*	bisphenol AF	0.149 or 1.487 (0.05 or 0.5 °)	4	↓	Rao et al. [209]
O	*D. rerio*	methylparaben	0.007 or 0.072 (1 or 11 *)	30	↓	Thakkar et al. [210]
O	*D. rerio*	ethanol	108.535 (5 °)	7–28	–	Agostini et al. [211]
O	*D. rerio* embryos	bisphenol A	49.936 (11.4 °)	1	↓	Murugan et al. [212]
O	*D. rerio* embryos	methylparaben	0.0007 or 0.007 (0.1 or 1 *)	6	↓	Raja et al. [213]
O	*D. rerio* embryos	octocrylene	0.014 (5 *)	4	–	Gayathri et al. [214]
			0.138 or 1.383 (50 or 500 *)		↓	
O	*D. rerio* embryos	benzophenone-3	0.001 or 0.010 (1 or 10 *)	3	↓	Sandoval-Gío et al. [215]
O	*D. rerio* embryos + larvae	ammonia NH_3_	3.523–49.912 (0.06–0.85 °)	7	↓	Mariz et al. [216]
O	*D. rerio* larvae	hexabromobenzene	0.054 (30 *)	6	–	Chen et al. [217]
			0.181–0.544 (100–300 *)		↓	
O		pentabromobenzene	0.063–0.212 (30–100 *)		–	
			0.635 (300 *)		↓	
O	*G. affinis*	bisphenol A	20.763 or 33.904 (4.74 or 7.74 °)	15–60	↓	Belhamra et al. [218]
O	*G. affinis*	decabromodiphenyl ether	0.026 or 0.052 (25 or 50 *)	2	↑	Pérez-Iglesias et al. [219]
O	*O. mossambicus*	ammonia NH_3_	58.720 (1 °)	28–56	↓	Gopi et al. [220]
O	*O. mossambicus*	dichloromethane	8595–9301 (730–790 °)	4	↓	Nirmala et al. [221]
O	*O. niloticus*	benzylparaben	2.19 × 10^−5^–0.022 (0.005–5 *)	56	↓	Lin et al. [222]
O	*O. niloticus*	guanitoxin	(125 or 250 “”)	4	↓	Passos et al. [223]

GCS—group of chemical substance; E—metals and other elements; F—fungicides; H—herbicides; I—insecticides; M—microplastics; N—nanoparticles; O—other substances; Pd—pharmaceuticals and disinfectants; Exp. dur.—exposure duration; d—days; n/a—not applicable; NPs—nanoparticles; *A. testudineus*—*Anabas testudineus*, *A. nobilis*—*Aristychthys nobilis*, *C. umbla*—*Capoeta umbla*, *C. auratus gibelio*—*Carassius auratus gibelio*, *C. punctatus*—*Channa punctatus*, *C. mrigala*—*Cirrhinus mrigala*, *C. gariepinus*—*Clarias gariepinus*, *C. decemmaculatus*—*Cnesterodon decemmaculatus*, *C. macropomum*—*Colossoma macropomum*, *C. idella*—*Ctenopharyngodon idella*, *C. carpio*—*Cyprinus carpio*, *D. rerio*—*Danio rerio*, *D. labrax*—*Dicentrarchus labrax*, *G. affinis*—*Gambusia affinis*, *H. fossilis*—*Heteropneustes fossilis*, *H. molitrix*—*Hypophthalmichthys molitrix*, *J. multidentata*—*Jenynsia multidentata*, *O. mykiss*—*Oncorhynchus mykiss*, *O. mossambicus*—*Oreochromis mossambicus*, *O. niloticus*—*Oreochromis niloticus*, *O. javanicus*—*Oryzias javanicus*, *O. latipes*—*Oryzias latipes*, *P. lineatus*—*Prochilodus lineatus*, *R. quelen*—*Rhamdia quelen*, *T. nilotica*—*Tilapia nilotica.*

### 3.2. The Effects of Toxic Agents on Acetylcholinesterase Activity

Analysis of the results shown in Table 3 revealed that in most cases (64%), AChE inhibition was reported; in 25% of cases, there was no change; and only in 11% of cases did an increase in AChE activity occur. In cases where no significant change observed, most data (44%) concerned lower concentrations of agents, while at higher concentrations, changes occurred; in 35% of cases, no change was a single result for one concentration tested or observed at all levels of a studied agent, and in 18% of cases, after shorter times of exposure. In contrast, at longer times, changes were observed, and only in 3% of cases (one case) did a nonlinear reaction occur, and no change was reported at the intermediate concentration of an agent. These data showed apparent concentration- and time-related effects of chemicals on AChE activity and a high responsiveness and sensitivity of this enzyme to various toxic agents. This confirms that AChE is a valuable neurotoxicity biomarker.

The results of many studies indicate that various aquatic pollutants may modulate AChE activity in fish, e.g., organophosphorus and carbamate insecticides are well-known AChE inhibitors that act by specifically binding to the active site of AChE and blocking the access of the physiological substrate [4,126,224,225], as well as organophosphate esters, used as plasticizers and flame retardants [226]. However, chemicals other than carbamates and organophosphates have also been documented to alter acetylcholinesterase activities (Table 3). In vitro inhibition of *Cyprinus carpio* AChE by 35 insecticides and their derivatives was tested [227]. It was found that various chemical forms of active compounds (e.g., oxon vs. thiono or diethyl vs. dimethyl) exhibited different inhibitory potencies. Also, combinations of different insecticides showed an additive inhibitory effect [227]. An in vitro study of the impact of metal ions on *Diodon hystrix* brain AChE activity [228] revealed inhibition order: Cr^6+^ < Co^2+^ < Ag^2+^ < Cu^2+^ < Pb^2+^ < As^5+^ < Cd^2+^ < Zn^2+^ < Ni^2+^ < Hg^2+^; and proved that AChE activity is a valuable biomarker for evaluating metal toxicity to fish.

AChE inhibitors include irreversible and reversible ones [126]. Reversible inhibitors, competitive or noncompetitive, mostly have therapeutic applications, while toxic effects are associated with irreversible AChE activity modulators [126].

The data of various studies showing the effects of aquatic pollutants on the fish brain or whole-body AChE activity (Table 3) reveal that most examined agents, including microplastics, pharmaceuticals, pesticides, metals, etc., usually inhibited the enzyme. Still, the effects were concentration- and time-related. AChE activation also sometimes occurred, or non-linear alternate bidirectional changes were observed at various concentrations or different times of exposure to the same agent, making interpretation of the neurotoxic effect difficult. In most cases of microplastic exposures, inhibition of AChE was observed; however, at low concentrations, enzyme activation sometimes occurred [135,138]. Also, most fish exposure to pharmaceuticals resulted in AChE inhibition, except for some antidepressants (sertraline) that induced no changes or activated the enzyme. The different effects observed in fish are related to the duration of exposure and the concentration of the pharmaceutical [152,196]. Most pesticides, such as insecticides, herbicides, fungicides, and antifouling agents, caused AChE inhibition in fish, often concentration- and/or time-related. Many commonly used triazine, carbamate, organophosphate, neonicotinoid, methylurea, or phenylurea pesticides are AChE inhibitors due to their binding to the AChE active site [229]. For some pesticides that usually inhibited AChE, activation was reported in single cases [155,186,208], which might have been related to the very low concentrations used. Inhibition of AChE activity by other chemicals was also reported for: disinfectants, bisphenol A, cosmetic compounds, organic solvents, metals and other elements, nitrogenous metabolites, and other aquatic pollutants. It is worth mentioning that, in the case of fish exposure to nanoparticles, AChE activity was rarely inhibited (Table 3), suggesting that environmentally relevant concentrations of these pollutants exhibit low neurotoxicity. According to the review by Olivares-Rubio and Espinosa-Aguirre [230], polycyclic aromatic hydrocarbons (e.g., benzo[a]pyrene, pyrene, and anthracene) inhibited AChE activity. Still, PAHs with a low molecular weight did not induce changes in AChE activity.

Acetylcholinesterase activity is sometimes measured in various tissues, and the results are not always the same as for the brain. They also differ among the organs examined. Different values and time- and concentration-related splenic and cardiac AChE activity patterns were observed in *Oncorhynchus mykiss* treated with clothianidin (a neonicotinoid insecticide) [231]. No alterations in brain AChE activities were reported following TiO_2_ and nano-TiO_2_ exposures, but an increase occurred in the gill and liver [147]. Different patterns of gill and muscle AChE activity changes over time of exposure to three pesticides, compared with the brain, were also reported [186]. No changes in brain AChE activity were observed following low and high cypermethrin or chlorpyrifos exposures, while muscle AChE activities significantly decreased after exposures to high concentrations of both pesticides [122]. AChE activity in the brain and muscles of three cyprinid fish species exposed to diafuran similarly decreased [162]. A considerable and significant decrease in AChE activity in the muscle of trichlorfon-exposed *Oreochromis niloticus* was also reported [232]. Similar changes in the brain and muscle AChE activities were observed in *Prochilodus lineatus* exposed to Roundup [2] and in *Danio rerio* treated with sulfoxaflor [172]. The decrease in AChE activities measured in the muscle, liver, and gill of *Oreochromis mossambicus* exposed to dichloromethane followed the decline in the brain [221]. However, the control value of AChE activity and the difference between the values for the control and exposed fish were the highest in the brain and muscle. The highest AChE activity was reported in the brain and muscle of *Anabas testudineus*. Enzyme inhibition following exposure to naphthalene also occurred in the gill and liver [203]. Chlorpyrifos reduced *Danio rerio* muscle AChE activity in a concentration-dependent manner. In contrast, no such relationship was observed for copper—a significant decrease in AChE activity occurred only at the lowest Cu concentration (6.3 µg/L) [233]. Similar activities and inhibition of brain and muscle AChE were reported in *Tor putitora* collected from various sampling sites with different pollution levels [234]. Some results showed an even higher sensitivity of muscle AChE, compared with the brain, to nano-Ag intoxication. These data indicate that AChE activity in muscle is probably an equally reliable biomarker as brain or whole-body AChE activity [149]. On the other hand, brain and muscle AChE may sometimes show opposite responses to toxic agents. In *Leiarius marmoratus* and *Pseudoplatystoma reticulatum* hybrids (pintado da Amazônia), AChE activity decreased in muscle but increased in the brain compared with the control group after Roundup exposure [235]. AChE activity in various tissues of *Danio rerio* was inhibited almost simultaneously when the fish were exposed to high concentrations of toxic agents (Cd^2+^ or deltamethrin). In contrast, at lower concentrations, inhibition occurred with a delay relative to the brain: brain > gill > muscle > liver [236]. These data suggest that muscle AChE activity should be interpreted with caution. Moreover, caution is also necessary because, for some toxic agents, AChE may not serve as a reliable biomarker of neurotoxicity. For example, a study conducted on *Danio rerio* revealed no changes in brain AChE activity during treatment with a 0.5% ethanol solution, while a significant decrease in acetylcholine level and choline acetyltransferase activity was observed [211].

Inhibition of AChE activity is usually associated with oxidative stress. Experimentally induced oxidative stress—caused by exposure of *Danio rerio* embryos to H_2_O_2_—resulted in a considerable and significant decrease in AChE activity [237]. However, the role of oxidative stress in modulating AChE activity remains unclear. According to various studies, oxidative stress may be involved in both the decrease [238,239] and increase [240] of AChE activity.

### 3.3. Intergenerational Effects of Toxic Agents on Acetylcholinesterase Activity

Although limited data are available, they indicate that AChE activity can serve as a biomarker of reproductive and intergenerational toxic effects, likely due to the parental transfer of neurotoxic compounds via gametes to the offspring. Larvae of *Danio rerio* obtained from adults exposed to a herbicide formulation containing 2,4-dichlorophenoxyacetic acid (2,4-D) exhibited elevated AChE activity [155]. In contrast, inhibition of AChE activity was observed in the offspring of *Danio rerio* adults exposed to rhodamine B dye [241]. It was also observed [242] that parental *Danio rerio* exposure to microcystin caused a reduction in dopamine, dihydroxyphenylacetic acid (DOPAC), and serotonin accompanied by hypoactivity and decreased AChE activity in the larvae. Microcystin was detected in the gonads of adults. It was probably transferred to the offspring, which affected the expression of genes involved in the neurotransmitter functions and neuronal development. It has also been reported that following parental exposure to benzo[a]pyrene, a significant reduction in AChE activity was observed in the larvae, along with decreased swimming velocity [116]. Genes involved in neuron growth and brain development were downregulated in the F1 larvae, while brain function in the exposed F0 adults remained unaffected. These findings suggest an epigenetic effect. A similar intergenerational epigenetic effect of chlorpyrifos-oxon on AChE activity has been observed [243]. Epigenetic mechanisms include acetylation or phosphorylation of histones, DNA methylation, and interactions of non-coding RNA during transcription. These may affect gene and protein expression [244] and suggest that neurotoxic compounds can be transferred via the gonads to the gametes and the offspring, causing developmental disturbances by changing gene expression. The effects observed in the next generation depend on the type of substance and the duration of exposure. Understanding the transgenerational impacts of environmental pollutants may help explain changes in species dynamics. Therefore, monitoring the effects of such pollutants is crucial as they may influence future generations and pose a threat to species survival.

### 3.4. Other Neurotoxicity Biochemical Biomarkers

In fish toxicology, other biochemical biomarkers of neurotoxicity are also occasionally used, such as the activities of BChE, PChE, and CbE, as well as levels of GABA (γ-aminobutyric acid); however, available data remain limited. Although GABA plays a key role as the primary inhibitory neurotransmitter in the central nervous system of fish, its use as a biomarker of neurotoxicity is associated with several significant limitations. GABA levels can be influenced not only by neurotoxic substances but also by general environmental stress, metabolic changes, temperature fluctuations, oxidative stress, and other forms of physiological disturbance [245,246]. Therefore, such changes may be nonspecific and do not always clearly indicate damage to the nervous system [245,246]. Monoamines such as dopamine, norepinephrine, and serotonin are valuable biochemical markers for assessing subtle neurotoxic effects in fish. However, like GABA, their diagnostic value increases when analyzed in conjunction with behavioral responses, enzymatic activities, and transcriptomic parameters. In *Cyprinus carpio* exposed to the dithiopyridine herbicide, concentrations of the neurotransmitters dopamine, norepinephrine, and acetylcholine were evaluated, revealing a decrease in catecholamine levels. In contrast, acetylcholine (ACh) concentration increased, accompanied by AChE inhibition [117]. In zebrafish exposed to methamphetamine, a concentration- and time-dependent decrease in brain levels of dopamine, norepinephrine, and serotonin was observed [118]. According to a meta-analysis by Santana et al. [247], fish cholinesterases (ChEs) are commonly used as biomarkers of environmental contamination due to their high sensitivity to a broad range of toxicants, including organophosphate and carbamate pesticides. The authors concluded that BChE response was more variable than AChE regardless of tissue, and no difference between their average activities was detected. Other authors emphasize the need to conduct species- and tissue-specific studies to characterize esterases before selecting them for use in monitoring programs [248]. The BChE enzyme is mainly associated with plasma or nonspecific tissue compartments, and in *Solea senegalensis* it shows minimal activity in relevant tissues such as muscle [249]. This limits its utility as a broad or sensitive biomarker across species and tissues. It was reported that malathion inhibited AChE and BChE activities in Senegalese sole (*Solea senegalensis*) in a dose- and time-dependent manner. Still, BChE activity was approximately two orders of magnitude lower than that of AChE [249].

## 4. Limitations of the Presented Studies

In this article, we have aimed to present the results of significant, well-designed, and carefully conducted scientific studies. However, it should be noted that these studies also have certain limitations related to the adopted conventions and methodologies.

Scientists conducting research on fish living in their natural habitats must contend with the dynamic changes occurring in the environment. Even slight variations in water temperature or oxygenation can strongly influence fish behavior as well as their motor and metabolic activity. The concentration of toxic substances in the environment can change significantly within just a few hours. Moreover, these substances exist in various chemical forms and engage in numerous interactions with both chemical and physical factors—factors that can also interact with one another. The half-lives of different xenobiotics in the environment vary and are influenced by physicochemical conditions.

The results of ecophysiological studies conducted in the natural environment provide a very accurate reflection of the physiological condition of fish living in waters with varying levels of pollution. However, it is not possible to determine the relationship between the dose or concentration of xenobiotics in the environment and their physiological effects based on the results of such studies.

The experimental regime used in laboratory studies allows for very precise determination of the physiological effects caused by specific doses of xenobiotics. However, a major limitation of this type of research is that experimental conditions often differ significantly from those found in natural environments. In many such studies, the doses of xenobiotics used are much higher than those typically encountered by fish in natural water bodies. Moreover, laboratory experiments often lack many of the environmental factors present in natural waters that can either neutralize toxic substances or enhance their toxicity.

Additionally, individual experiments often differ in key conditions that are fundamental to fish physiology, such as ammonium (NH_3_) concentration, nitrate (NO_3_^−^) and nitrite (NO_2_^−^) level, total and carbonate hardness, salinity, dissolved oxygen concentration, pH, temperature, and so on. Furthermore, in some publications, authors do not provide precise information about the physicochemical conditions under which the experiments were conducted.

The dose/physiological effect relationship is primarily influenced by the chosen experimental model, the method of chemical administration, and the duration of exposure. Different fish species exhibit varying sensitivities to xenobiotics, and, in general, substances administered by injection tend to produce a stronger physiological effect than those absorbed from water or ingested with food. Table 2 and Table 3 provide summaries that allow for a more detailed analysis of this issue.

To illustrate the mechanisms of neurotoxicity, we have cited the results of several experiments conducted under in vitro conditions. In vitro techniques typically involve cell cultures that do not account for interactions with bioregulators present in the organism or with other cells that together form tissues and organs. While the findings of such studies are scientifically valuable, they should be interpreted with great caution when extrapolating to conditions within the organism—and even more so when extrapolating to those in the natural environment.

## 5. Conclusions

Most aquatic pollutants cause oxidative stress in fish, the symptoms of which are elevated levels of oxidation products and changes in activities and concentrations of the components of the endogenous antioxidant system. Oxidative stress is a primary mechanism of tissue damage and physiological disorders. AChE activity is a sensitive and valuable but nonspecific biomarker of neurotoxicity in fish. It can be affected by many aquatic pollutants that mainly cause enzyme inhibition, and the responses are concentration- and time-related. This results in disturbed acetylcholine hydrolysis, the consequences of which are disturbed sensory, cognitive, and motor functions. Many aquatic pollutants show oxidative and neurotoxic potential, including developmental toxicity, which is a considerable threat not only to fish but also to their consumers and users of contaminated water: wildlife, domestic animals, and humans. Therefore, the inclusion of oxidative stress parameters and AChE activity measurements in routine natural water quality biomonitoring and toxicological studies is recommended. However, such assessments in toxicological experiments should be conducted under standardized experimental conditions, including defined physicochemical water parameters and appropriate selection of model organisms and tested tissues.

## 6. Recommendations

The analysis we conducted while preparing this review article led us to formulate a set of recommendations which, we hope, may serve as guidance for other researchers. Our recommendations are as follows:

(1) In toxicity studies of various chemicals, it is essential—following ethical considerations and recommendations—to include biochemical parameters, such as indicators of oxidative stress and AChE activity.
(2) In toxicological experiments, the potential influence of the physicochemical parameters of water on the results of biochemical analyses should be taken into account. It is important that researchers measure and report individual water quality parameters, such as the concentrations of ammonia, nitrites, and nitrates.
(3) The vast majority of toxicological experiments focus on the effects of a single substance on fish of a given species. There is a need for studies aimed at determining the effects of co-exposure to multiple toxicants (e.g., pesticides and heavy metals).
(4) There are few studies that include more than one fish species. Comparative studies involving various fish species simultaneously are needed.
(5) It is recommended to conduct toxicological studies taking into account various development stages of fish (embryos, larvae, fry, sexually mature individuals).
(6) Detailed information on the mechanisms of action and physiological roles of cholinesterases other than AChE in fish is lacking. Further research is needed to address this knowledge gap.

## Figures and Tables

**Table 1:** Products of oxidative damage of biological molecules that are used as biomarkers of oxidative stress in fish.

Biomarker	Process	References
lipid peroxides	oxidative damage to lipids	Tanaka et al. [15]
hydroxyl lipids	oxidative damage to lipids	Baker et al. [16]; Tanaka et al. [15]
malondialdehyde	oxidative damage to lipids	Liu et al. [17]; Paital et al. [14]; Sánchez-Nuño et al. [18]; Safari Asl et al. [19]; Tan et al. [20]
4-hydroxynonenal	oxidative damage to lipids	Topal et al. [21]; Sánchez-Nuño et al. [18]
lipofuscin	oxidative damage to lipids	Pulster et al. [22]; Raibeemol and Chitra [23]; Zhong et al. [24]; Gómez Manrique et al. [25]; Ottinger et al. [26]
8-hydroxy-2′-deoxyguanosine, 8-oxo-7,8-dihydro-2′-deoxyguanosine	oxidative damage to DNA guanosine	Mahboub et al. [27]; Jia et al. [28]; Mohamed et al. [29,30]; Topal et al. [31]
8-hydroxyguanine	oxidative damage to DNA guanine	no data found
5,6-dihydroxy-5,6-dihydrothymine	oxidative damage to DNA thymine	no data found
advanced oxidation protein products	oxidation of proteins	Sánchez-Nuño et al. [18]
protein carbonyls	oxidation of proteins	Almeida et al. [32]; Zhang et al. [33]; Blank do Amaral et al. [34]; Iturburu et al. [35]; Feng et al. [36]
Hydroxyleucine	oxidation of protein leucine	no data found
Nitrotyrosine	nitration of protein tyrosine	Shahab et al. [37]; Lee et al. [38]

**Table 2:** The effects of toxic agents on oxidative stress parameters commonly determined in fish toxicology. The original values are given in parentheses and expressed in the following units: ° mg/L * µg/L ** µmol/g (injection) ^~^ µL/L ^ µmol/kg (injection) ^^ mg/kg ^#^ ng/L ^##^ ppm ^###^ mg/kg (injection).

	Fish Species	Toxic Agent	Exp. dur. [d]	Concentration [µmol]	No Change	Decrease	Increase	Reference
Ah	*Anabas testudineus*	naphthalene	3	32.789 (4.2 °)	CAT, GPx, GSH, TBARS	–	–	Nayak et al. [66]
				34.329 (4.4 °)	TBARS	GPx, GSH	CAT, TBARS	
				35.890 (4.6 °)	–	GPx, GSH	CAT, TBARS	
				37.450 (4.8 °)	–	GPx, GSH	CAT, TBARS	
				39.010 (5 °)	–	GPx, GSH	CAT, TBARS	
Ah	*Carassius auratus*	benzo(k)fluoranthene	1–15	0.399 (0.1 ^^)	GSH, GST, TBARS	–	–	Ji et al. [67]
				0.240 (0.06 ^^)	GST, TBARS	GSH	–	
				1.189 (0.3 ^^)	TBARS	GSH	GST	
				5.870 (1.48 ^^)	TBARS	GSH	GST, TBARS	
				29.367 (7.41 ^^)	–	GSH	GST, TBARS	
Ah	*Clarias gariepinus*	benzene	30	9.76 × 10^−6^ (0.762 ^#^)	GST	SOD, TAS	MDA	Sayed et al. [68]
Ah	*Clarias gariepinus*	toluene	30	0.00033 (26.614 ^#^)	GST, SOD, TAS, MDA	–	–	Sayed et al. [68]
Ah	*Clarias gariepinus*	xylene	30	0.0008 (89.403 ^#^)	GST, SOD, TAS, MDA	–	–	Sayed et al. [68]
Ah	*Colossoma macropomum*	benzo[a]pyrene	4	(1 ^)	SOD, CAT, GPx, GST,	–	–	Sadauskas-Henrique et al. [69]
					LPO			
					SOD, CAT, GPx, GST			
				(10 ^)	SOD, CAT, GPx, GST	–	LPO	
				(100 ^)	CAT, GPx	–	SOD, LPO	
				(1000 ^)		–	SOD, GST, LPO	
Ah	*Dicentrarchus labrax*	polycyclic aromatic hydrocarbons	21	(835 ± 52 ^#^)	GSH, SOD, GPx, CAT	SOD	GSH	Danion et al. [70]
Ah	*Gambusia yucatana*	polycyclic aromatic hydrocarbons	4	(4.37 *)	SOD, GPx	TBARS, CAT	GST	Aguilar et al. [71]
				(8.73 *)	SOD, GPx,	TBARS, CAT	GST	
				(17.46 *)	SOD, GPx	TBARS, CAT	GST	
				(34.95 *)	GPx	TBARS, CAT	GST, SOD	
E	*Dicentrarchus labrax*	mercury (as HgCl_2_)	4	0.037 (0.010 °)	–	–	LPO	Barboza et al. [72]
				0.059 (0.016 °)	–	–	LPO	
E	*Oreochromis mossambicus*	selenium (as Na_2_SeO_3_)	4	0.029 (5 *)	–	–	LPO, GSH	Gobi et al. [73]
				0.058 (10 *)	–	–	LPO, GSH	
				0.145 (25 *)	–	–	LPO, GSH	
				0.289 (50 *)	–	–	LPO, GSH	
				0.578 (100 *)	–	–	LPO, GSH	
F	*Carassius auratus*	Topas 100 EC	4	5.278 (1.5 °)	LOOH, SOD, CAT, GPx,	–	–	Husak et al. [74]
					GST, GR			
					LOOH, CAT, GST, GR			
				52.783 (15 °)	LOOH, GR	GST	SOD, GPx, GST	
				87.972 (25 °)		GST	SOD, CAT, GPx, GST, GR	
F	*Chanos chanos*	carbendazim	4	0.015 (2.85 *)	LPO, CAT, GST	–	–	Palanikumar et al. [75]
				0.029 (5.45 *)	LPO	CAT, GST	–	
				0.057 (10.97 *)	–	CAT, GST	LPO	
				0.105 (20.17 *)	–	CAT, GST	LPO	
				0.237 (45.31 *)	–	CAT, GST	LPO	
F	*Danio rerio*	azoxystrobin	7–28	0.002 (1 *)	GST, MDA	SOD, CAT	ROS, SOD, GST, MDA	Han et al. [76]
				0.025 (10 *)	CAT, GST, MDA	SOD	ROS, SOD, CAT, GST, MDA	
				0.248 (100 *)	CAT, GST	SOD	ROS, SOD, CAT, GST, MDA	
F	*Oncorhynchus mykiss*	propiconazole	4	14.727 (5.04 °)	TBARS, CAT, GR	SOD, CAT, GPx, GR	SOD, GPx, TBARS	Li et al. [77]
H	*Astyanax altiparanae*	atrazine (as Atrazina Atanor^®^)	30	0.002 (0.5 *)	CAT, SOD, MDA	GST	–	Destro et al. [78]
				0.005 (1 *)	CAT, SOD, MDA	GST	–	
				0.009 (2 *)	CAT, SOD, MDA	GST	–	
				0.046 (10 *)	SOD, MDA	GST	CAT	
H	*Clarias batrachus*	pretilachlor	30–60	0.930 (0.29 °)	GR	–	TBARS, SOD, CAT	Verma et al. [79]
		(50% EC)		1.251 (0.39 °)	GR	–	TBARS, SOD, CAT	
				1.860 (0.58 °)	GR	–	TBARS, SOD, CAT	
H	*Clarias gariepinus*	oxyfluorfen	60	3.207 (1.16 °)	–	SOD, CAT, GPx, GSH	MDA	El-Houseiny et al. [80]
H	*Colossoma macropomum*	glyphosate (as Roundup)	4	59.146 (10°)	SOD, GPx	GST	–	Braz-Mota et al. [81]
				88.719 (15 °)	SOD	GST	GPx	
H	*Cyprinus carpio*	fenaxoprop-P-ethyl	15–30	0.104 (37.5 *)	–	GPx, GR, GST	MDA	Neglur et al. [82]
H	*Danio rerio*	atrazine	14	0.005 (1 *)	MDA, SOD, CAT	GSH	–	Jin et al. [83]
				0.046 (10 *)	MDA	GSH	SOD, CAT	
				0.464 (100 *)	CAT	GSH	SOD, MDA	
				4.636 (1000 *)	–	GSH	SOD, CAT, MDA	
H	*Labeo rohita*	glyphosate	4–12	2.957 (0.5 °)	ROS, TBARS, GSH, CAT,	–	–	Ghaffar et al. [84]
					SOD, POD			
				3.549 (0.6 °)	ROS, TBAR, GSH, CAT,	–	–	
					SOD, POD			
				4.140 (0.7 °)	–	GSH, CAT, SOD, POD	ROS, TBARS	
				4.732 (0.8 °)	–	GSH, CAT, SOD, POD	ROS, TBARS	
H	*Oreochromis*	glyphosate (as Roundup)	14–28	3.549 (0.6 °)	–	GSH	MDA	Abdelmagid et al. [85]
	*niloticus*							
H	*Oreochromis niloticus*	glyphosate-based herbicide	14	(5 °)	–	–	–	Acar et al. [86]
				(10 °)	–	–	CAT	
				(20 °)	–	TBARS	CAT	
				(30 °)	–	TBARS	CAT	
				(40 °)	–	TBARS	CAT	
H	*Rhamdia quelen*	atrazine	4	0.046 (10 *)	GPx, MDA	CAT		Gomes et al. [87]
I	*Carassius gibelio*	deltamethrin	1–14	0.004 (2 *)	GPX	SOD,CAT, GPX, GST, GR, GSH	LPO, CAT, GPX, GST, GR, GSH	Dinu et al. [88]
I	*Chanos chanos*	chlorpyrifos (as Trickel)	4	0.004 (1.38 *)	LPO, CAT, GST	–	–	Palanikumar et al. [75]
				0.006 (2.15 *)	LPO	CAT, GST	–	
				0.013 (4.53 *)	–	CAT, GST	LPO	
				0.026 (9.27 *)	–	CAT, GST	LPO	
				0.054 (18.97 *)	–	CAT, GST	LPO	
I	*Channa punctatus*	malathion (commercial grade)	4–12	1.211 (0.4 °)	–	–	SOD, CAT, LPO	Bharti and Rasool [89]
I	*Colossoma macropomum*	malathion (emulsion)	4	22.097 (7.30 °)	SOD, GPx, LPO	–	GST, CAT, SOD, GPx	Souza et al. [90]
I	*Cyprinus carpio*	malathion (commercial formulation)	14	1.514 (0.5 °)	–	GPx, GSH	MDA, SOD, CAT	Yonar [91]
				3.027 (1 °)	–	GPx, GSH	MDA, SOD, CAT	
I	*Cyprinus carpio*	malathion (commercial formulation)	10	1.514 (0.5 °)	–	GPx, GSH	MDA, SOD, CAT	Yonar et al. [92]
				3.027 (1 °)	–	GPx, GSH	MDA, SOD, CAT	
I	*Clarias batrachus*	chlorpyrifos	15	4.706 (1.65 °)	–	SOD, CAT, GSH, LPO, GST, GPX	SOD, CAT, GSH, LPO, GST, GPX	Narra et al. [93]
		(20% EC)						
I	*Clarias batrachus*	monocrotophos	15	9.590 (2.14 °)	–	SOD, CAT, GSH, LPO, GST, GPX	SOD, CAT, GSH, LPO, GST, GPX	Narra et al. [93]
		(36% EC)						
I	*Clarias gariepinus*	imidacloprid (as Sunclopride 35% SC)	60	0.008 (2.03 *)	–	SOD, GPx	MDA	Abdel Rahman et al. [94]
I	*Cyprinus carpio*	fipronil (as Standak-BASF)	8	0.001 (0.65 *)	MDA, GST	–	MDA, GST	Menezes et al. [95]
I	*Cyprinus carpio*	profenofos	60	0.013 (4.74 *)	–	SOD, CAT, GSH	MDA	Abdel Rahman et al. [96]
I	*Cyprinus carpio*	quinalphos	20	0.0009 (1.09 ^~^)	–	–	SOD, CAT, MDA, GST	Hemalatha et al. [97]
		(25% EC)		0.0018 (2.18 ^~^)	–	–	SOD, CAT, MDA, GST	
I	*Piaractus mesopotamicus*	endosulfan (as Zebra Ciagro^®^)	4	0.003 (1.1 *)	–	–	GPx, CAT, GST, GR	Bacchetta et al. [98]
I	*Piaractus mesopotamicus*	lambda-cyhalothrin (Cilambda^®^)	4	0.016 (0.7 *)	–	GR, GST	CAT, GPx, GR, GST	Bacchetta et al. [98]
I	*Rhamdia quelen*	fipronil (as Standak-BASF)	8	0.002 (0.65 *)	MDA, GST	GST	MDA	Menezes et al. [95]
M	*Cyprinus carpio*	polystyrene	21	(1000 ^#^)	–	CAT, SOD, GPX	ROS, MDA	Cui et al. [99]
		microplastics						
M	*Dicentrarchus labrax*	fluorescence red polymer microspheres	4	(0.26 °)	–	–	LPO	Barboza et al. [72]
				(0.69 °)	–	–	LPO	
M	*Oreochromis niloticus*	microplastics (>100 nm in size)	15	(1 °)	–	TAC	SOD, CAT, TPX	Hamed et al. [100]
				(10 °)	–	TAC	SOD, CAT, TPX,	
				(100 °)	–	TAC	SOD, CAT, TPX	
O	*Ctenopharyngodon idella*	ammonium acetate	n/a	9000 (9 **)	CAT	SOD, GSH,	MDA	Xing et al. [101]
O	*Ctenopharyngodon idella*	bisphenol A	14	0.014 (3.2 ^##^)	–	CAT, GSH	TBARS, GST	Faheem and Lone [102]
O	*Cyprinus carpio*	bisphenol A	30	13.141 (3.0 °)	TBARS, ROS	–	–	Afzal et al. [103]
				19.712 (4.5 °)	–	SOD, CAT, POD, GSH	TBARS, ROS	
				26.282 (6 °)	–	SOD, CAT, POD, GSH	TBARS, ROS	
O	*Oreochromis*	methyl tert-butyl ether	27	0.028 (2.5 ^~^)	–	TAO	SOD, CAT, GPx, MDA	Banaee et al. [104]
	*niloticus*			0.057 (5 ^~^)	–	GR, TAO	SOD, CAT, GPx, MDA	
O	*Pelteobagrus fulvidraco*	ammonium acetate	n/a	8000 (8 **)	GPX	TAO	SOD, CAT, MDA	Zhang et al. [105]
O	*Sparus aurata*	bisphenol A	21	21.902 (5 ^^^^)	GST	–	CAT	Maradonna et al. [106]
				219.020 (50 ^^^^)	CAT, GST	–	–	
O	*Oreochromis mossambicus*	diisononyl phthalate	1–4	0.717 (300 ^##^)	SOD, CAT, GR, GPx	SOD, CAT, GPx, GR	SOD, CAT, GR, MDA	Revathy and Chitra [107]
Pd	*Carassius gibelio*	gentamicin	3	3.595 (5 ^###^)	GSH, SOD, CAT, GPx	–	–	Bojarski et al. [108]

Exp. dur.—exposure duration; d—days; n/a—not applicable; CAT—catalase; GPx—*g*lutathione peroxidase;** GR—glutathione reductase; GST—glutathione *S*-transferase; GSH—reduced glutathione; LPO—lipid peroxidation; MDA—malondialdehyde; POD—peroxidase; SOD—superoxide dismutase*;* ROS—reactive oxygen species; TAO—total antioxidant; TBARS—thiobarbituric acid reactive substances; TPX—total peroxides; Ah—aromatic hydrocarbons; E—elements; F—fungicides; H—herbicides; I—insecticides; M—microplastics; O—other substances.

## Data Availability

The dataset supporting the reported results can be found at scientific databases, e.g., Web of Science or PubMed.

## Abbreviations

The following abbreviations are used in this manuscript:

2,4-D	2,4-dichlorophenoxyacetic acid
8-OHdG	8-hydroxy-2′-deoxyguanosine
8-OHGua	8-hydroxyguanine
8-oxodG	8-oxo-7,8-dihydro-2′-deoxyguanosine
*A. nobilis*	*Aristychthys nobilis*
*A. testudineus*	*Anabas testudineus*
ACh	Acetylcholine
AChE	Acetylcholinesterase
Ah	Aromatic hydrocarbons
BChE	Butyrylcholinesterase
*C. auratus gibelio*	*Carassius auratus gibelio*
*C. carpio*	*Cyprinus carpio*
*C. decemmaculatus*	*Cnesterodon decemmaculatus*
*C. gariepinus*	*Clarias gariepinus*
*C. idella*	*Ctenopharyngodon idella*
*C. macropomum*	*Colossoma macropomum*
*C. mrigala*	*Cirrhinus mrigala*
*C. punctatus*	*Channa punctatus*
*C. umbla*	*Capoeta umbla*
CAT	Catalase
CbE	Carboxylesterase
ChEs	Cholinesterases
Cu,ZnSOD	Copper-zinc superoxide dismutase
CYPs	Cytochrome P450 enzymes
d	Days
*D. labrax*	*Dicentrarchus labrax*
*D. rerio*	*Danio rerio*
DNA	Deoxyribonucleic acid
DOPAC	Dihydroxyphenylacetic acid
E	Elements
EROD	Ethoxyresorufin-O-deethylase
Exp. dur.	Exposure duration
F	Fungicides
*G. affinis*	*Gambusia affinis*
GABA	γ-aminobutyric acid
GCS	Group of chemical substance
GPx	Glutathione peroxidase
GR	Glutathione reductase
GSH	Reduced glutathione
GSSG	Oxidized glutathione
GST	Glutathione S-transferase
H	Herbicides
*H. fossilis*	*Heteropneustes fossilis*
*H. molitrix*	*Hypophthalmichthys molitrix*
I	Insecticides
*J. multidentata*	*Jenynsia multidentata*
LPO	Lipid peroxidation
m	Minutes
M	Microplastics
MDA	Malondialdehyde
MFOs	Mixed-function oxygenases
MnSOD	Manganese superoxide dismutase
MTs	Metallothioneins
N	Nanoparticles
n/a	Not applicable
NMDA receptor	*N*-methyl-D-aspartate receptor
NPs	Nanoparticles
NRF2	Nuclear factor erythroid 2-related factor 2
O	Other substances
*O. javanicus*	*Oryzias javanicus*
*O. latipes*	*Oryzias latipes*
*O. mossambicus*	*Oreochromis mossambicus*
*O. mykiss*	*Oncorhynchus mykiss*
*O. niloticus*	*Oreochromis niloticus*
*P. lineatus*	*Prochilodus lineatus*
PChE	Propionylcholinesterase
POD	Peroxidase
*R. quelen*	*Rhamdia quelen*
ROS	Reactive oxygen species
SOD	Superoxide dismutase
*T. nilotica*	*Tilapia nilotica*
TAO	Total antioxidant
TBARS	Thiobarbituric acid reactive substances
TPX	Total peroxides
